# The effect of shoulder prosthesis stem length on failure due to torsional loading. A biomechanical study in composite humeri

**DOI:** 10.1016/j.jseint.2023.04.011

**Published:** 2023-05-24

**Authors:** Weston K. Ryan, Wyatt D. Vander Voort, Maarouf A. Saad, Edward Wu, Tanya C. Garcia-Nolen, Christopher O. Bayne, Robert M. Szabo

**Affiliations:** a Department of Orthopaedics, University of California, Davis, Sacramento, CA, USA; b Department of Orthopaedic Surgery, University of Minnesota, Minneapolis, MN, USA

**Keywords:** Shoulder, Arthroplasty, Periprosthetic, Fracture, Biomechanics, Sawbones, Torsion

## Abstract

**Background:**

Shoulder arthroplasty is becoming increasingly common. With evolving implant designs, multiple humeral stem options exist for the surgeon to choose from. New stemless and short-stem systems are modular, remove less native bone stock, and better adapt to patient anatomy. It has been suggested that shorter stem implants may be protective against periprosthetic fracture; however, this has not been mechanistically evaluated. Therefore, this study aimed to biomechanically test synthetic humeri with long-stem, short-stem, and stemless arthroplasty components in a torsional manner to evaluate their response to loading and characterize failure.

**Methods:**

Twenty-four synthetic humeri were implanted with long stem, short stem, or stemless uncemented prosthesis, 8 in each group. Humeri were mounted in a custom testing jig with a morse taper interfacing with a mechanical testing system. After a 20N axial force, specimens were torsionally loaded to failure at 15 degrees/sec, with 50 Hz collection. Torque vs. rotation curves were generated for each specimen, and stiffness, yield, ultimate strength, and failure load were measured. ANOVA and post hoc pairwise comparisons were used to assess effect of stem type on mechanical test variable. The association of the stem type with fracture type was analyzed by a Fisher’s Exact test. Statistical significance was set at *P* < .05.

**Results:**

During torsional loading, long-stem implants were significantly stiffer than short or stemless implants. The angle of implant yielding was similar across stem designs; however, stemless implants had a lower yield torque. This correlated with a decreased yield energy in stemless compared to short stems as well. Maximum torque and failure torque was also significantly higher in short-stem and long-stem implants compared to stemless.

**Discussion:**

Periprosthetic fractures in shoulder arthroplasty are a concern in low-energy trauma, and stem design likely plays a significant role in early implant-bone failure. Our results suggest stemless implants under torsional load fail at lower stress and are less stiff than stemmed implants. The failure mechanism of stemless implants through metaphyseal cancellous bone emphasizes the effect bone quality has on implant fixation. There is likely a balance of torsional stability to survive physiologic loads while minimizing diaphyseal stress and risk of diaphyseal periprosthetic fracture. This combined with revision and fixation options represent decisions the surgeon is faced with when performing shoulder arthroplasty.

Both anatomic and reverse shoulder arthroplasty has seen a rapid increase in volume and incidence. In 2002, the per capita rate of shoulder arthroplasty in the United States was 24.5 per 100,000. By 2011 the rate had increased to 54.4 per 100,000 with 66,485 total procedures performed.^32^ This increase in volume can be related to an aging population, improved implant design, introduction of the reverse total shoulder (rTSA), and expanding indications.^22^

The humeral component in shoulder arthroplasty has undergone significant evolution. The first cohort of shoulder arthroplasty patients presented by Neer et al in 1974 utilized long-stem humeral monoblock components that relied on cement fixation.^27^ Second generation components increasingly relied on bony ingrowth, although the ability to recreate native anatomy was still limited by humeral head and neck options.^7^^,^^29^ Third generation components are increasingly modular, allowing the surgeon to more accurately recreate anatomy with options such as stems with variable neck shaft angles and offset humeral heads.^16^ The trend in modern humeral components is towards shorter-stemmed metaphyseal and stemless implants with options available from most major manufactures.^19^ The primary benefit of short-stem and stemless humeral components in shoulder arthroplasty is the preservation of bone from decreased stress shielding, osteolysis associated with a longer stem, and ease of revision. It has also been suggested that short-stem and stemless components may be protective against fracture^5^; however, the specific effect is not known.

Periprosthetic fractures of the humerus have been reported at rates from 0.5% to 3.0% and represents up to 20% of all complications in shoulder arthroplasty.^6^^,^^23^^,^^30^ Periprosthetic fractures around long stem prostheses are most commonly spiral fractures with a high rate of component loosening, most often requiring surgical stabilization or revision arthroplasty.^2^^,^^8^^,^^11^^,^^17^^,^^24^^,^^33^^,^^34^ These fractures are associated with decreased shoulder function, long healing times or nonunion, high rates of reoperation, and complications such as radial nerve palsy and deep infection.^8^^,^^24^ Differences in postoperative periprosthetic fracture risk specifically between anatomic and rTSA are difficult to quantify and poorly studied.^28^

The presence of a long-stem prosthesis is known to create a stress riser that predisposes to fracture.^11^ Additionally, intramedullary reaming has been demonstrated to further weaken bone and evidence of overzealous reaming is frequently observed when reviewing periprosthetic fractures.^11^ Lee et al demonstrated that when reaming in preparation for placement of a humeral implant, bone is typically removed asymmetrically leading to more bone removal than necessary, exacerbating the problem of the stress riser.^25^ One of the proposed benefits of a short stem or a stemless humeral implant is elimination of the stress riser, reducing the risk of fracture.^19^ Currently there is limited literature to validate the hypothesis that a stemless implant is protective against periprosthetic fracture when compared to a traditional longer stem implant, especially under torsional loads that are involved in spiral fracture types. Jones et al performed a biomechanical investigation of the analogous hypothesis in periprosthetic femur fractures and demonstrated that synthetic femora implanted with short-stem arthroplasty components tolerated significantly greater torsional forces before fracturing than those implanted with long stem arthroplasty components.^21^ Similarly, recent literature has evaluated torsional loads on synthetic humeri evaluating micromotion. However, failure mechanics under torsional loading has yet to be evaluated and would offer novel insight into shoulder arthroplasty implant behavior and failure mechanics.^14^ The semiconstrained nature of reverse total shoulder arthroplasty (rTSA) has been theorized to impart increased torsional forces on the humeral construct.^14^ Additionally, the humeral stem may be subjected to predominately torsional forces at end-range-of-motion, or under conditions of impingement. Together with the high incidence of spiral periprosthetic fracture patterns, torsional loading was believed to be a pertinent failure mode for study in rTSA implants.

Here, the authors performed a biomechanical investigation in which synthetic humeri implanted with uncemented long-stem, short-stem, and stemless arthroplasty components were subjected to torsional force until sustaining a fracture. The aim of this study was to investigate the impact of humeral stem length on resistance to fracture under torsional loading and the resulting fracture pattern. We hypothesized that decreasing stem length would yield a lower torsional ultimate strength, as well as alter the failure mechanism.

## Materials and methods

### Component selection, implantation, and mechanical testing

To compare the effects of the different stem lengths, long-stem, short-stem, and stemless humeral prostheses were implanted into synthetic humeri. These humeri were then subjected to torsional mechanical testing. Synthetic humeral bones were chosen to allow for consistent methodology and comparison between cohorts. The chosen model (Sawbones Model #3404-4) is left sided and intended to mimic mildly osteoporotic bone. These are dual density, constructed of a foam composite and polyurethane cortical shell, have previously been validated for use in biomechanical models, and have been used to study humeral stem torsional motion.^4^^,^^14^^,^^15^^,^^18^ Twenty-four total humeri (8 long stem, 8 short stem, and 8 stemless) were implanted and tested.

Three uncemented implants were chosen for comparison: a long-stem prosthesis, a short-stem prosthesis, and a stemless prosthesis (Wright Medical Group N.V., Memphis, TN, USA). The long and short-stemmed implants have matching proximal geometry intended to match metaphyseal geometry. They are constructed with a coarse hydroxyapatite coating proximally and a fine coated distal section of variable length. The long stem length measures 93 millimeters (mm) and the short stem length measures 70 mm. Both are implanted utilizing analogous instrumentation and technique. The broaches utilized are intended to impact instead of remove bone and neither requires reaming. The stemless component utilized relies on 3 hydroxyapatite coated fins for fixation.

**Figure 1:**
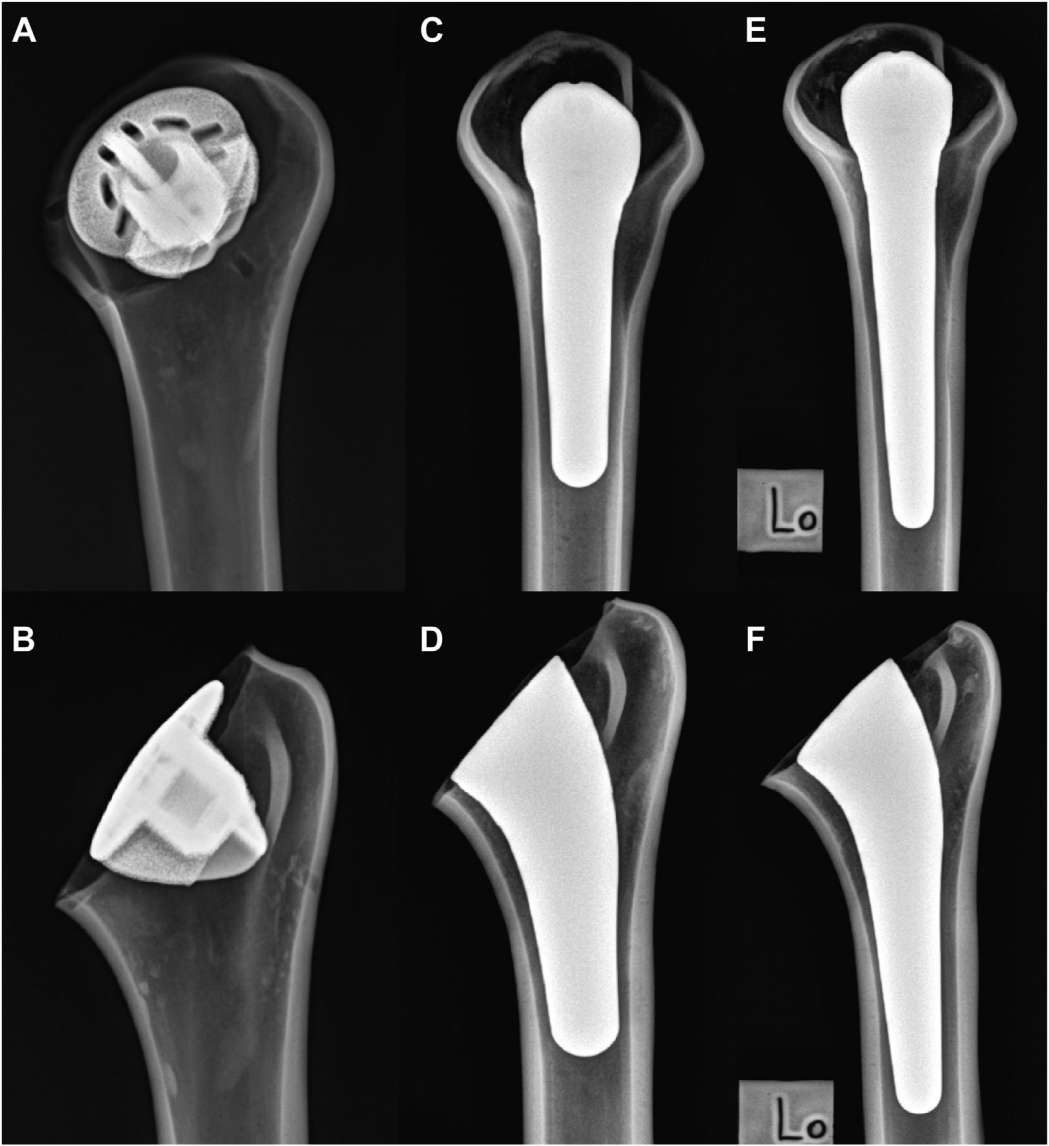
Lateral and anteroposterior radiographs of stemless (**A**, **B**), short-stem (**C**, **D**), and long-stem (**E**, **F**) implants.

Initial humeral preparation and component sizing was performed by an experienced shoulder surgeon (R.M.S.) and verified by visual inspection, angled cutting guides, and radiographs (NEXT digital radiography system; Sound, Carlsbad, CA, USA; 56 kVp, 2.0 mAs) for appropriate implant position. The technique and instrumentation used for humeral preparation is consistent with that used in the operative setting. We began by pinning the humeral cutting guide to the humerus at the level of the anatomic humeral neck performing the humeral head osteotomy with an oscillating saw. The inclination of the humeral head cut was measured relative to the medullary canal and found to be 132.5°. The humeral canal was then prepared in accordance with the described technique sequentially utilizing the provided sounders and impaction broaches. A standard sizing guide was utilized to determine the component as a size 2b (Size 2 diameter for both the long and short stemmed prosthesis, b = neck shaft angle of 132.5) and a centralized pin was placed for humeral preparation. The same humeral cutting method was utilized in implantation of the stemless component. After humeral preparation, the final implant was placed and inspected, and final radiographs obtained (Fig. 1). Slight differences in neck cuts were observed as the cutting guide was secured independently in all specimens in an attempt to recreate realistic intraoperative preparation and implantation. However, the head-neck angle was confirmed equal in all specimens and other small variability was deemed acceptable for this testing protocol.

**Figure 2:**
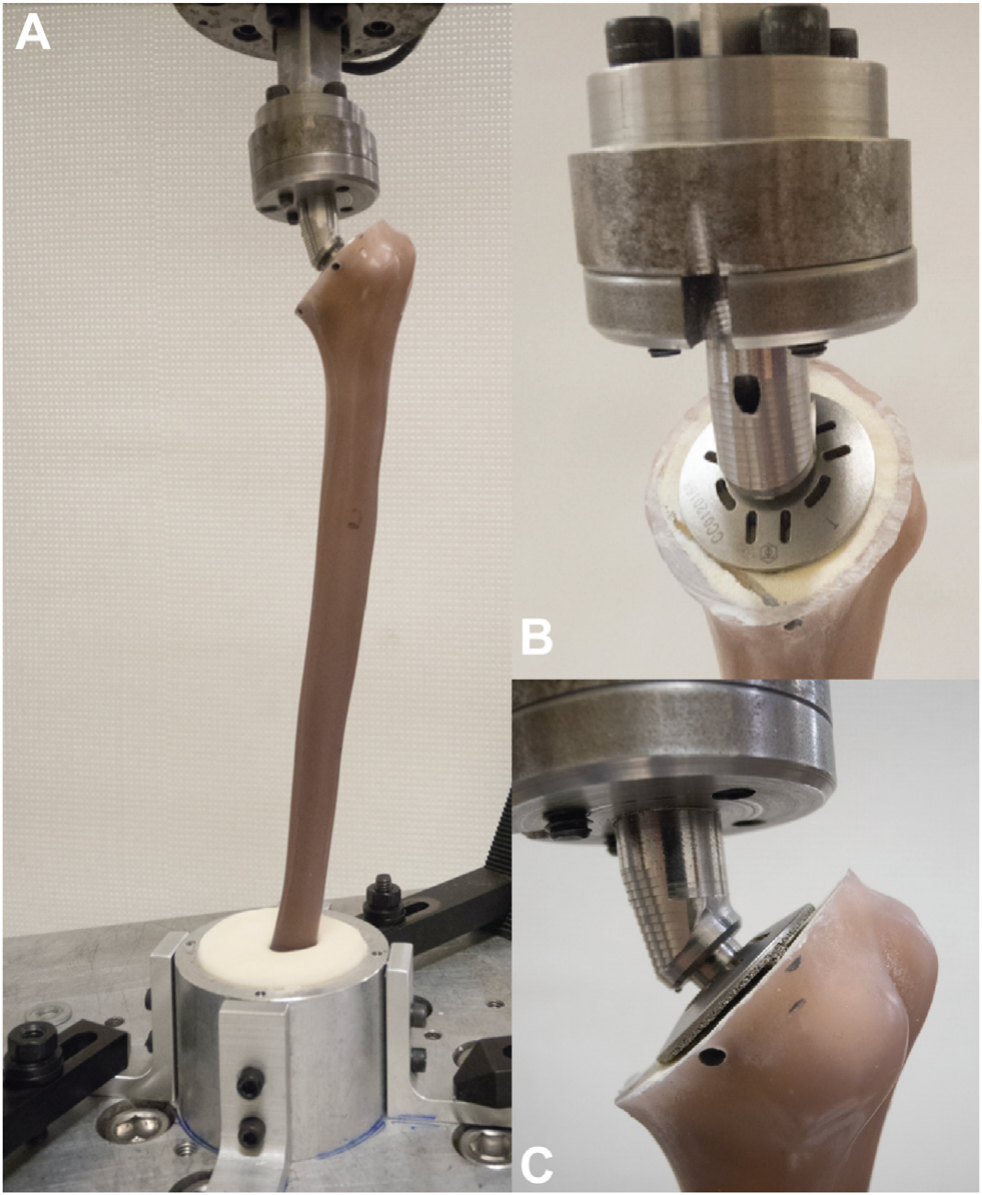
Clinical photos showing potted humerus assembled in testing jig (**A**), and custom morse taper torsional adapter on a stemless implant (**B**, **C**).

The center of the distal end of the humerus was aligned with the center of rotation platform of the machine. The distal-most 7.5 cm of the humerus was potted in polymethylmethacrylate (PMMA; Coe-Tray Plastic, GC America, Chicago, IL, USA) in a custom cylindrical cup and affixed to the platform. This allowed for secure mounting to the servohydraulic testing machine (STM) in accordance with a previously described “potting” technique.^4^ Care was taken to align the specimen with the long axis of the humerus vertically oriented in the sagittal and coronal plane during potting. The metal cylinder was mounted to the base of the servohydraulic testing machine with the long axis of the humerus in line with the servohydraulic crosshead in order to minimize bending forces during torsional loading. Humeral and implant position was monitored during loading for nonrotational deformation that may alter the torsional vector at the implant interface. The crosshead was securely attached to the proximal end of the specimen through an adapter that interfaced with the morse taper on the prosthesis. A custom adapter with a morse taper (2.4 deg taper angle) that mimics the morse taper on the backside of a humeral head final implant engaged into the implant at an angle of 140 deg to the vertical. This adapter was built to allow fixation of the humeral models to the mechanical test system (Model 662.20C01; MTS Systems, Eden Prairie, MN, USA). The testing device with humeral component can be seen in Figure 2.

The humeri were loaded axially to 20N compression to maintain a stable construct and avoid component loosening, then tested to failure in torsion by external rotation of the distal end 80 deg at 15 deg/sec. This testing protocol has been previously described in torsional testing of composite humeri.^15^ Video was synchronously recorded with a 60 Hz video camera (S-PRI; AOS Technologies AG, Dättwil, Switzerland). This protocol was chosen because the most common mechanism of fracture is a low-energy fall resulting in a spiral fracture.^8^^,^^11^^,^^13^^,^^24^ Testing was performed on a machine that simultaneously records force and displacement.

### Data analysis

**Figure 3:**
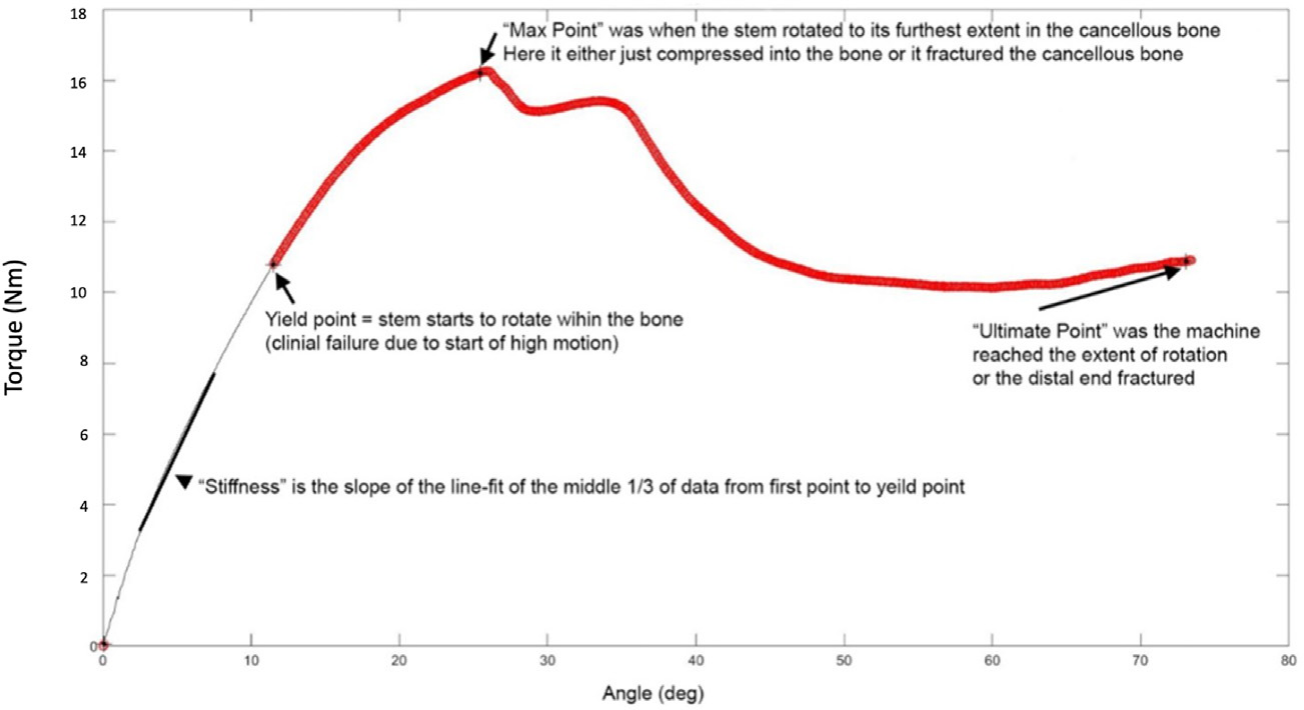
Torsional stress-strain curve example of a short-stem implant test with defined points of interest.

Throughout testing, torque, axial load, and vertical and rotational displacement were measured with a sampling rate of 50 times per second. Torsion vs. rotation curves were generated for each load test. Stiffness was calculated as the slope from the middle third of the data from start of loading to the yield point. Yield was defined as the point when the rotation intercept of the least-squares linear fit of the torque-rotation curve had a 1% or greater offset from the prior data point’s intercept. The maximum point was defined as the point of maximum torque, and the failure point was the point at either 80 deg or where torque dropped to 0 (fracture). Torque, rotation, and energy (torque-rotation integral) were captured at yield, maximum and failure points. All data were analyzed using custom software (Matlab; The Mathworks, Natick, MA, USA). The parts of the curve corresponded to occurrence in the bone as shown in Figure 3.

### Statistical analysis

The effect of stem type on mechanical test variables was assessed using a repeated measures ANOVA (SAS Institute, Cary, NC, USA) and post hoc pairwise comparisons. Normality of the ANOVA residuals was assessed using the Shapiro–Wilk statistic. When residuals were not normally distributed for a variable, a repeated measures ANOVA was performed on the rank-transformed variable. The association of the stem type with fracture type was analyzed by a Fisher’s Exact test. Statistical significance was set at *P* < .05.

## Results

**Figure 4:**
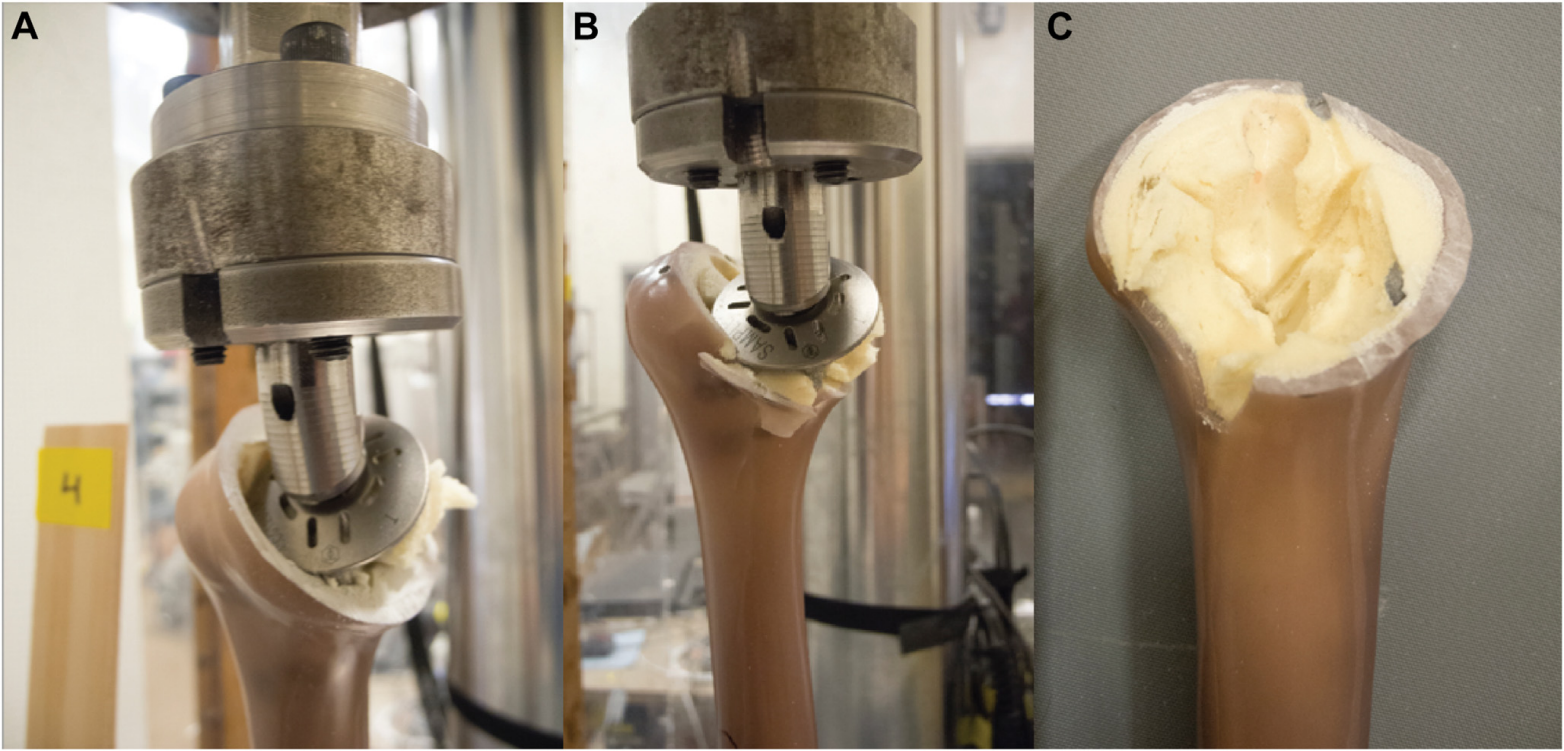
Representative humerus after stemless implant failure demonstrating cancellous metaphyseal failure (**A**, **B**) and short fracture extension into medial cortex (**C**).

**Figure 5:**
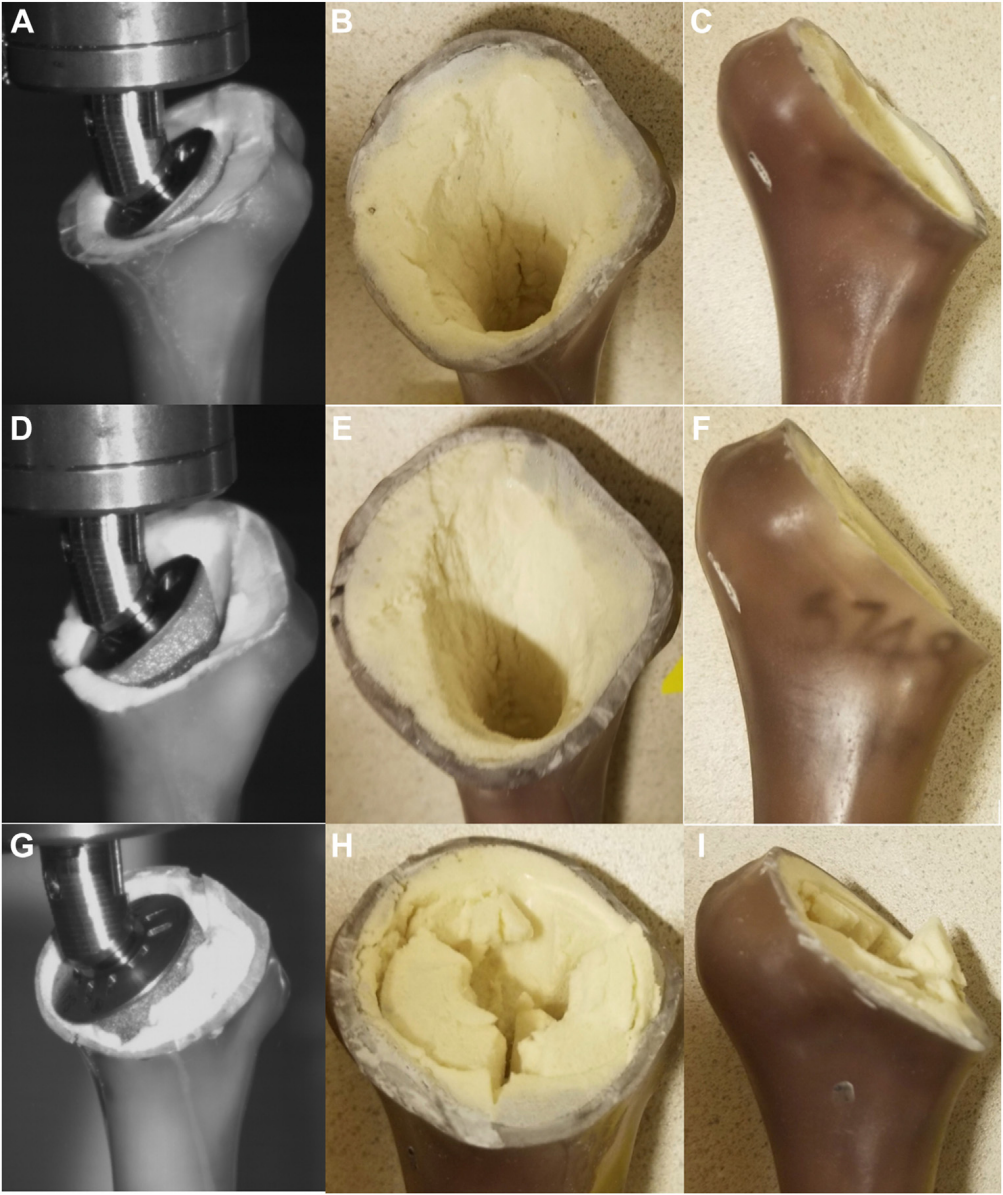
Long-stem (**A**-**C**), short-stem (**D**-**F**), and stemless (**G**-**I**) models at the time of failure and after explantation.

All specimens failed at the bone-implant interface. In 5 cases (3 long-stem, 1 short-stem, and 1 stemless), a short fracture also extended through the medial proximal metaphyseal cortex but did not propagate distally. Mode of failure was not statistically significant. Figure 4 demonstrates an example of a fracture through the medial proximal metaphyseal cortex in a stemless model. Images of long-stem, short-stem, and stemless models at the time of failure as well as after explantation can be seen in Figure 5. Throughout testing, no discernable bending was observed of the humeral shaft in any specimen, indicating a predominately torsional force and minimal off-axis bending moment relative to the humeral shaft stiffness.

**Figure 6:**
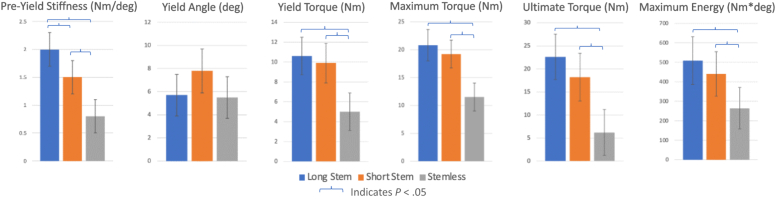
Graphical representation of preyield stiffness, yield angle, yield torque, maximum torque, ultimate torque, and maximum energy.

Long-stem implants demonstrated significantly higher preyield stiffness compared to short-stem and stemless implants, and short stem implants were significantly stiffer than stemless implants. Both long and short-stem implants demonstrated significantly higher yield torque, maximum torque, maximum energy, and ultimate torque than stemless implants. There was no significant difference in yield angle, maximum angle, or ultimate angle between the 3 implants. Results are summarized in Table I and Figure 6.

**Table I:** Biomechanical properties of long stem, short stem, and stemless humeral implants.

Variable	Prosthesis [Mean (SD)]			Difference (*P* value)		
	Long	Short	Stemless	Long vs. short	Long vs. stemless	Short vs. stemless
Pre-Yield Stiffness (Nm/deg)	2.0 (0.3)	1.5 (0.3)	0.8 (0.3)	**.009**	**<.001**	**.002**
Yield Angle (deg)	5.7 (1.8)	7.8 (1.9)	5.5 (1.8)	.099	.868	.073
Yield Energy (Nm∗deg)	35 (14)	44 (14)	19 (14)	.431	.053	**.011**
Yield Torque (Nm)	10.6 (1.9)	9.9 (2.0)	5.0 (1.9)	.602	**<.001**	**.001**
Max Angle (deg)	34.4 (6.9)	33.0 (6.4)	30.9 (6.0)	.751	.426	.621
Max Torque (Nm)	20.8 (2.8)	19.2 (2.5)	11.5 (2.5)	.381	**<.001**	**<.001**
Max Energy (Nm∗deg)	509 (122.7)	440.5 (113.9)	264.6 (106.6)	.405	**.006**	**.029**
Ultimate Angle (deg)	69.8 (10.2)	64.0 (11.0)	75.4 (10.3)	.433	.424	.128
Ultimate Torque (Nm)	22.6 (4.9)	18.2 (5.2)	6.2 (5.0)	.219	**<.001**	**.003**

*SD*, standard deviation; *Nm*, newton-meter; *deg*, degrees.

Bold values are statistically significant

## Discussion

Total shoulder arthroplasty and rTSA are both becoming increasingly common procedures in the United States.^32^ Between 2011 and 2017, the number of shoulder replacements increased by 103.7%, and future growth rates are predicted to outpace both total knee and total hip arthroplasty.^31^ During this dramatic rise, an increasing prevalence of complications has been observed. Periprosthetic fractures represent a complex problem for surgeons, oftentimes necessitating revision surgery.

Short-stem and stemless humeral components provide numerous proposed benefits.^19^ First, these implants preserve proximal humeral bone stock, allowing for increased fixation options in the setting of a periprosthetic fracture. Theoretically, revision surgery is technically less challenging with these implants given the increased bone stock and the ease of removal. However, each fracture location presents unique considerations.^28^ For example, a metaphyseal fracture may have increased comminution but allow for treatment with a long revision stem, while a diaphyseal fracture may be less likely to result in stem loosening but require a larger surgical approach. These implants may also limit stress shielding as more metaphyseal bone is loaded. In addition, metaphyseal stems avoid a diaphyseal stress riser under bending loads. A diaphyseal stress riser in long stem implants may also result in part from reaming of the canal. Prior studies have demonstrated that both eccentric and excessive reaming in preparation for placement of a humeral implant may increase the risk of fracture, predominately under bending stress where the implant tip or reaming creates a stress riser.^11^^,^^25^ Finally, in complex congenital or post-traumatic deformity, these implants allow for more options to reconstruct the proximal humerus without relying on the anatomic axis between the diaphysis and the humeral head.^19^

Despite the multiple proposed benefits of short-stem and stemless implants, there is little evidence to support some of these claims, particularly under torsional loading. We present, to our knowledge, the first biomechanical study evaluating specific torsional failure mechanics of humeral arthroplasty components. In this study, when an external rotation force was applied to the 3 implant constructs, differences in ultimate torque, energy, and stiffness were all observed. This highlights the significant mechanical differences that implant design can impart. In all models, failure occurred at the sawbone-implant interface within the cancellous bone of the proximal humeral metaphysis in what we describe as the implant “scooping out” of the bone. An insignificant number of small fractures extended through the medial cortex of the proximal humerus. There were no fractures at the tip of stemmed implants involving the humeral diaphysis. This may be in part due to no bending force being applied on the model. It also may be a result of weakness at the sawbone-implant interface as would be seen in the early postoperative period. *In vivo*, bony ingrowth would strengthen this interface over time and influence mechanical testing. Ingrowth on longer stem implants that are significantly torsionally stiffer at baseline would further transfer torsional loads to the distal diaphyseal cortical segment, which may propagate spiral fracture patterns in high enough loads. This effect is not well-studied. The full biomechanical effect of a long stem compared to short stem during failure would best be evaluated in a system that could account for ingrowth/ongrowth, which our present study could not test.

Stemless implants have been shown to be most suited for patients with adequate bone stock, and the results of our study support this conclusion in a torsional model. Chen et al demonstrated increased ultimate strength of stem-based implants in a model comparing bone mineral density, supporting the use of longer stems in patients with osteoporotic bone.^12^ Our results align with these conclusions. The mode of failure on all stemless implants involved failure of metaphyseal cancellous bone, again highlighting the importance of metaphyseal bone quality for initial stemless implant stability. As metaphyseal “scoop-out” occurred even in a rigid Sawbones model, stemless implants are less likely to transmit significant torsional load to the diaphysis than longer stems and may be protective against distal spiral periprosthetic fractures. This comes at the theoretical risk of early component loosening or excess motion to impede ingrowth. However, there has been repeatable evidence of stemless implants in anatomic arthroplasty models having good survivability and sufficient resistance to early micromotion under physiologic loads, challenging the clinical significance of the increased stability stemmed implants offer and illustrating sufficient clinical stability of stemless implants.^1^^,^^3^^,^^9^^,^^10^^,^^14^^,^^26^ Longer term follow-up on new stemless rTSA implants that will likely see introduction into US markets will reveal if these designs show equal survivorship, though little is published regarding fracture. Nonetheless, this study illustrates failure mechanisms in multiple humeral stem implants and can be cautiously applied to the more common stemless constructs used in anatomic arthroplasty, especially when evaluating torsional failures.

Stemless implants demonstrated significantly lower yield torque compared to short and long stems. This correlated with a decreased yield energy in stemless compared to short stems as well. Therefore, stem-type implants appear to allow for more energy absorption in the event of a fall. However, this also may indicate higher torque transmission to the distal segment of a long stem. As higher preyield stiffness was seen with increasing stem length, the engagement of the additional distal stem length carries the added load. The pattern of results in this study while using stemmed implants without significant distal diaphyseal engagement (such as long revision-type implants) indicates the effect a small increase in stem length could potentially have on mechanical properties. This work adds to the evidence that stemless implants do not appear to have the same propensity for diaphyseal periprosthetic fractures as stemmed implants, though bone quality may have a significant role in surgeon implant choice as evidenced by the mechanism of failure. Ultimately, there is a balance of arthroplasty component stability (ie, longer stem, bone quality) with surgical factors such as ease of implantation and morbidity in a revision scenario.

This study is not without its limitations. First, synthetic humeri were used for the biomechanical testing. While offering a uniform testing medium, the precise hoop stress mechanics of the implanted Sawbone humeri has not been well studied, particularly with respect to impaction or broaching. This may lead the absolute values of our study to differ from results obtained in cadaveric specimens. However, there remains value of comparative testing between stem lengths as fourth generation sawbones have been validated as an appropriate *in vitro* model for testing the biomechanical properties of the humerus as well as torsional motion.^4^^,^^14^^,^^15^^,^^18^ Additionally, sawbones have been used in the evaluation of torsional stability of femoral stems with similar results to cadaveric models.^20^ Another limitation of this study is that the custom device used to induce an external rotation force and cause periprosthetic fractures does not accurately represent all mechanisms of injury and the internal and external forces that are applied to the proximal humerus during these injuries, and cannot account for soft tissue or scapulothoracic interplay. There is a complex combination of axial, bending, and torsional forces that vary widely with the mechanism of injury, degree of implant stability, patient position, and bone quality. Native shoulder torque has been shown to reach over 30 Nm even in nonathletes, suggesting even with significant soft-tissue mitigation of an externally applied load to the shoulder, implants could see loads above the failure threshold.^35^ In terms of methodology, another limitation is the slight variation within implant position and neck cuts as the authors utilized the system cutting guide in an effort to accurately recreate intraoperative use. The neck cuts were confirmed at an equal angle, and the small variation in position was thought to be insignificant. Finally, a future study using cadaveric specimens with stem fixation to simulate ingrowth as well as revision-type diaphyseal implants would further expand our current understanding of the role of humeral stem length in these injuries beyond early stability.

## Conclusion

The current biomechanical study used a sawbones model to evaluate fracture characteristics and failure mechanics in stemless, short-stem, and long-stem shoulder arthroplasty components under a torsional load. While anatomic humeral implants were utilized due of the lack of availability of stemless reverse arthroplasty implants in the US at the time of this study, the results of this study may be particularly relevant in cases of rTSA given the constrained motion and loading mechanics. Stiffness increased with increasing stem length. Maximum torque and failure torque was also significantly higher in short and long-stem implants compared to stemless implants. Stemless implants appear to be less torsionally stable which indicates a susceptibility to failure in poor metaphyseal bone. However, if there is sufficient stability for physiologic loading until boney ingrowth and implant incorporation, then this may correlate with a lower risk for spiral diaphyseal periprosthetic fracture. A larger study reviewing early periprosthetic humerus fractures with varying stem length and fracture pattern is warranted to further investigate the clinical scope of this work. Overall, this study evaluates novel biomechanical behavior of various shoulder arthroplasty implants which provides surgeons with information that can guide component selection for their patients.

## Disclaimers

Funding: No funding was disclosed by the authors.

Conflicts of interest: Dr. Bayne is a paid consultant for Lima Corporate. The other authors, their immediate families, and any research foundation with which they are affiliated have not received any financial payments or other benefits from any commercial entity related to the subject of this article.
